# Anterior insular network disconnection and cognitive impairment in Parkinson’s disease

**DOI:** 10.1016/j.nicl.2020.102364

**Published:** 2020-07-25

**Authors:** Yasmine Y. Fathy, Dagmar H. Hepp, Frank J. de Jong, Jeroen J.G. Geurts, Elisabeth M.J. Foncke, Henk W. Berendse, Wilma D.J. van de Berg, Menno M. Schoonheim

**Affiliations:** a Department of Anatomy and Neurosciences, Amsterdam Neuroscience, Amsterdam UMC, Vrije Universiteit Amsterdam, Amsterdam, The Netherlands; b Department of Neurology, Amsterdam Neuroscience, Amsterdam UMC, Vrije Universiteit Amsterdam, Amsterdam, The Netherlands; c Department of Neurology, Erasmus Medical Center, PO Box 2040, 3000 CA Rotterdam, The Netherlands

**Keywords:** AIC, anterior insular cortex, ACC, anterior cingulate cortex, BC, betweenness centrality, dAI, dorsal anterior insula, DMN, default mode network, FC, functional connectivity, FPN, frontoparietal network, LP, Lewy pathology, PD, Parkinson’s disease, VENs, von Economo neurons, Insular cortex, Resting state fMRI, Cognitive impairment, Parkinson’s disease, Network triad

## Abstract

**Background:**

The insula is a central brain hub involved in cognition and affected in Parkinson’s disease (PD). The aim of this study was to assess functional connectivity (FC) and betweenness centrality (BC) of insular sub-regions and their relationship with cognitive impairment in PD.

**Methods:**

Whole-brain 3D-T1, resting-state functional MRI and a battery of cognitive tests (CAMCOG) were included for 53 PD patients and 15 controls. The insular cortex was segmented into ventral (vAI) and dorsal (dAI) anterior and posterior sub-regions. Connectivity between insular sub-regions and resting-state networks was assessed and related to cognition; BC was used to further explore nodes associated with cognition.

**Results:**

Cognitive performance was significantly lower in PD patients compared to controls (p < 0.01) and was associated with FC of the dAI with default mode network (DMN) (adjusted R^2^ = 0.37, *p* < 0.001). In controls, cognitive performance was positively related to FC of the dAI with the fronto-parietal network (FPN) only (adjusted R^2^ = 0.5, *p* = 0.003). Regionally, FC of the dAI with the anterior cingulate cortex (ACC) was significantly reduced in PD (F(1,65) = 11, *p* = 0.002) and correlated with CAMCOG (r = 0.4, *p* = 0.001). DMN and FPN showed increased BC in PD which correlated with cognition and reduced connectivity of dAI with the ACC (r_s_ = −0.33, *p* = 0.014 and r_s_ = −0.44, *p* = 0.001 respectively).

**Conclusions:**

These results highlight the relevance of the insula in cognitive dysfunction in PD. Disconnection of the dAI with ACC was related to altered centrality in the DMN and FPN only in patients. Disturbance in this network triad appears to be particularly relevant for cognitive impairment in PD.

## Highlights

• Cognitive dysfunction in PD is related to FC of the dorsal anterior insula (dAI)
• In PD only, FC between the dAI and DMN was most strongly related to cognition.
• FC of dAI with anterior cingulate was reduced and related to cognition in PD.
• Increased DMN and FPN centrality is related to dAI-ACC disconnection in PD.
• Altered interplay between dAI, DMN, and FPN underlies poor cognition in PD.

## Graphical abstract

**Figure:**
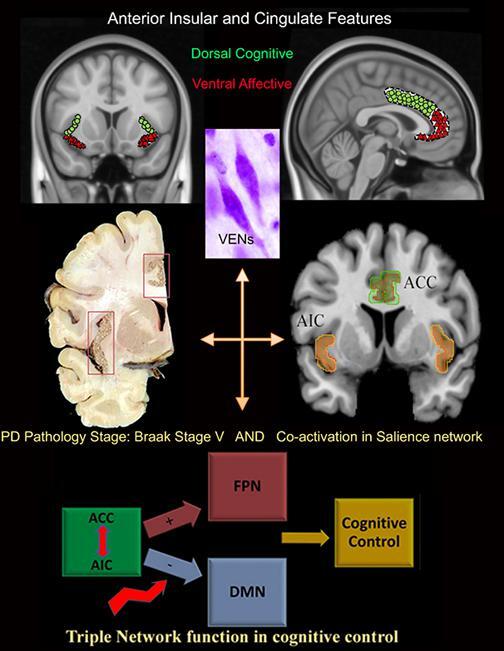

## 1 Introduction

Unlike motor symptoms, the pathophysiology of cognitive impairment in Parkinson’s disease (PD) is largely unknown and difficult to predict (Svenningsson et al., 2012). Mild cognitive impairment is already present in the prodromal phase of the disease in a subset of patients (Fengler et al., 2017) and in about 20% of patients at the time of clinical diagnosis (Aarsland, 2016). Eventually, with disease progression, most patients will develop dementia (Aarsland et al., 2003). Post-mortem brain studies indicated that cortical Lewy-body and β-amyloid pathology, cholinergic and noradrenergic deficits contribute to cognitive impairment in PD (Del Tredici and Braak, 2016, Irwin et al., 2012). Moreover, network studies show that connectivity changes within the default mode network (DMN) positively correlate with cognitive impairment in PD (Olde Dubbelink et al., 2014, Wolters et al., 2018). Notably, the DMN is influenced by the salience network, primarily composed of the anterior insula, indicative of the insula’s crucial role in cognition (Sridharan et al., 2008, Uddin et al., 2011).

The insular cortex is a central brain hub characterized by dense connections with the rest of the brain as well as dense internal connections, thus allowing and promoting efficient communication and integration of functions across the brain (van den Heuvel and Sporns, 2013).

The insular cortex is thought to orchestrate the function of cognitive networks such as the fronto-parietal network (FPN) and DMN (Menon and Uddin, 2010, Menon, 2011). Characterized by a complex cytoarchitecture, the insular cortex is sub-divided into the ventral anterior insula (vAI), involved in the processing of emotions and autonomic functions, the dorsal anterior insula (dAI), involved in cognitive control, and the posterior insula (PI), related to perception of internal states (Mesulam and Mufson, 1982). Each insular sub-region is characterized by a preferential set of connections, vAI to limbic regions, dAI to a range of limbic and cortical regions, and PI to auditory and somatosensory cortical regions, all sub-serving various functions (Mesulam and Mufson, 1982). Insular cortex atrophy and dopaminergic deficits have been shown to correlate with cognitive deficits in early-stage PD with mild cognitive impairment (Christopher et al., 2014, Christopher et al., 2014, Mak et al., 2014). Moreover, in post-mortem analysis, the anterior insula showed more severe pathology compared to the posterior insula in PD and PD with dementia (Fathy et al., 2019). Nevertheless, despite the surging evidence of the insula’s crucial role, it remains unclear how insula’s connectivity changes in PD may contribute to cognitive impairment.

The aim of this study was to analyze insular resting-state functional connectivity (FC) with resting-state networks, assess the importance of the three insular sub-regions within the entire brain network, and their relationship with cognitive performance in PD and controls. We hypothesized that disconnection of the anterior insula would be associated with cognitive impairment in PD.

## 2 Materials and methods

### 2.1 Participants:

This retrospective study included subjects from a previous study comprising sporadic PD patients and healthy age-matched controls recruited from our local movement disorders outpatient clinic (Olde Dubbelink et al., 2014). The research protocol was approved by the local institutional ethics review board, of the VU university medical center, in adherence to the Helsinki declaration. All participants provided written informed consents to participate in the study. Healthy volunteers were recruited as either spouses of patients or non-neurological participants within the same age range. PD patients fulfilled the UK Parkinson’s Disease Brain Bank Clinical diagnostic criteria (Hughes et al., 1992). Exclusion criteria in the original study for PD patients included previous stereotactic surgery, extensive white matter lesions as well as other abnormalities seen on structural MRI images. Subjects were examined by a trained neurologist and a radiologist with specific experience in movement disorders. Educational levels were classified according to the International Standard Classification of Education with scores ranging from 0 to 6 (0 = no primary education & 6 = university education). The cohort initially included data from 55 PD patients and 15 controls for whom complete clinical, neuropsychological, and imaging data were available. Two PD patients had insular hypo-intensities on 3D-T1 suspected of being infarcts and were therefore excluded, thereby bringing the final sample size to 53 PD patients. To avoid uncontrolled motion and reduce patient burden, all motor and cognitive tests as well as scanning were performed while patients were “ON” dopaminergic medication.

### 2.2 Neurological assessment

Age-of-onset and disease duration were calculated based on the first occurrence of PD motor symptoms as experienced by the patients. The Unified Parkinson’s Disease Rating Scale part III (UPDRS-III) was used to assess the severity of motor symptoms (Fahn, 1986), Hoehn and Yahr staging was used to provide information on patients’ disease stages (Hoehn and Yahr, 1967). PD patients were on combinations of anti-parkinsonian medications including L-DOPA, catechol-O-methyltransferase (COMT) inhibitors, monoamine oxidase inhibitors (MAO), dopamine agonists, and amantadine. An L-DOPA equivalent daily dose (LEDD) was calculated for each patient as previously described to standardize doses (Tomlinson et al., 2010) (Table 1).

**Table 1:** Subject Demographics. All test results, except Mini mental status exam, were significantly worse in PD compared to controls. Education is based on ISCED system ranging from 0 to 6. CAMCOG_Total: Cambridge Cognitive Battery Total score, H&Y: Hoehn and Yahr stage, ISCED: International Standard Classification of Education, LEDD: Levodopa equivalent daily dose, PD: Parkinson’s disease, SD: Standard Deviation, UPDRS III: Unified Parkinson’s Disease Rating Scale part III. * p < 0.05, ** p < 0.01.

Demographics and Functional Tests		Group				p values
		Controls (n = 15)		PD (n = 53)		
		N/count (%)	Mean (Range)	N/Count (%)	Mean (Range)	
Age (Years)		15	66,9 (52–81)	53	67,3 (54–81)	*p* = 0.85
Education	2	2 (13,3%)	–	16 (30,2%)	–	*p* = 0.07
	3	3 (20%)	–	15 (28,3%)	–	
	4	1 (6,7%)	–	2 (3,8%)	–	
	5	8 (53,3%)	–	19 (35,8%)	–	
	6	1 (6,7%)	–	1 (1,9%)	–	
Gender (Males)		66%	–	58%	–	*p* = 0.57
Disease duration (Years)		NA	NA	53	11,3 (4–21)	NA
UPDRS_III		NA	NA	53	32 (14–56)	NA
HY	2,0	NA	NA	21 (39,6%)	–	NA
	2,5	NA	NA	18 (34%)	–	NA
	3,0	NA	NA	14 (26,4%)	–	NA
Mini Mental Status Exam		14	28,3 (27–30)	53	27,6 (19–30)	*p* = 0.3
CAMCOG_Total		14	99,1 (95–104)	53	92,6 (71–103)**	*p* < 0.001
Semantic Fluency		14	24 (16–40)	53	19,5 (5–32)*	*p* = 0.03
Pattern Recognition Memory		14	22,9 (21–24)	52	20,6 (13–24)**	*p* < 0.001
Spatial span length		14	5,5 (5–7)	52	4,6 (2–8)*	*p* = 0.01
Spatial Working Memory		14	22,9 (5–57)	52	39,8 (2–106)*	*p* = 0.015
Intra-extra dimension shift		14	8,6 (7–9)	51	7,2 (0–9)**	*p* = 0.003
Vienna Perseveration Test		14	18,6 (14,3–27,5)	52	25,2 (10,2–63,2)*	*p* = 0.001
Beck Depression Inventory		14	4,8 (0–23)	52	12,2 (0–31)**	*p* = 0.001
Beck Anxiety Inventory		12	24,5 (21–31)	31	34,2 (24–57)**	*p* < 0.001
LEDD		NA	NA	53	1032 (225–2600)	NA

### 2.3 Cognitive and neuropsychiatric assessment

Cognitive performance was evaluated using the Mini Mental Status examination (MMSE) as well as the Cambridge Cognitive Examination revised test battery (CAMCOG). The latter assesses seven cognitive domains, namely: orientation (maximum score: 10), language (maximum score: 30), memory (maximum score: 27), attention and calculation (maximum score: 9), praxis (maximum score: 12), abstract thinking (maximum score: 8), and perception (maximum score: 9) (Huppert et al., 1995). Other cognitive tests included in the study consist of semantic fluency, pattern recognition memory (PRM), spatial span (SSP), spatial working memory (SWM), the intra-extra dimensional set shift test (IED), and the Vienna perseveration test (VPT). Furthermore, anxiety and depression were assessed using the Beck Anxiety Inventory (BAI, only available in a small sub-sample) and the Beck Depression Inventory (BDI) (https://beckinstitute.org/).

### 2.4 Image acquisition

All participants underwent structural whole-brain 3T MR imaging (MRI) (Sigma HDXT, V15M; GE Healthcare, Waukesha, Wis) using a sagittal three-dimensional T1-weighted fast spoiled gradient-echo sequence (repetition time msec/echo time msec, 7.8/3.0; inversion time msec, 450; flip angle, 12°; 1.0 × 0.9 × 0.9 mm voxel size). For resting-state functional MRI, 202 volumes of axial echo-planar images were acquired, of which the first two were discarded (TR1800/TE35; flip angle, 90°; 3.3-mm isotropic voxel size). Participants were asked to think of nothing in particular, close their eyes, and relax without sleeping during the functional MRI. Imaging was performed in the “ON” medication state as previously described (Olde Dubbelink et al., 2014, Hepp et al., 2017).

### 2.5 Definition of insular cortex sub-regions

**Fig. 1:**
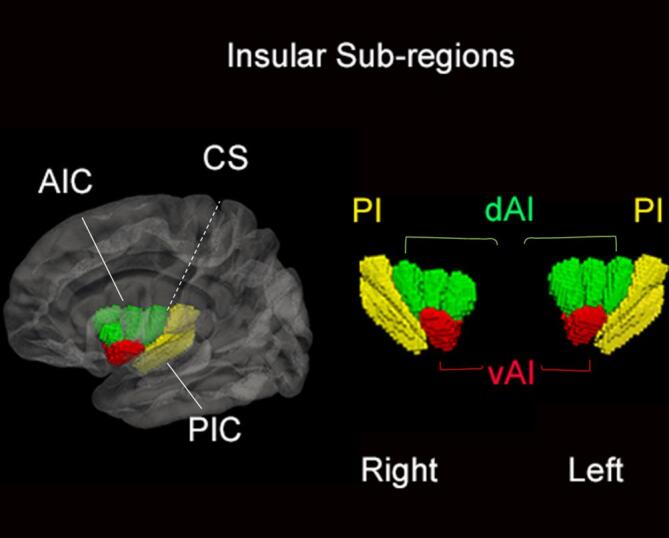
3D rendering of the insular sub-regions. The insular cortex is divided into anterior insular cortex (AIC), comprising ventral (vAI) and dorsal anterior insula (dAI), and a posterior insular cortex (PIC) separated by the central sulcus (CS).

Delineation of the insular cortex sub-regions was based on the Hammers_mith brain atlas, a stereotactic atlas based on manual segmentations of the insular cortex in MRI native space in 30 controls and summarized into a probabilistic map in MNI space (*http://soundray.org/hammers-n30r95*). The insula atlas featured six sub-regions within each hemisphere: anterior inferior cortex, anterior short gyrus, middle short gyrus, posterior short gyrus, anterior long gyrus, and posterior long gyrus (Faillenot et al., 2017). Based on the anatomical features of the insular cortex mentioned in previous literature (Naidich et al., 2004), we merged the sub-regions of the short gyrus into one region designated as the dorsal anterior insula (dAI). The anterior and posterior long gyri were merged as the posterior insula (PI) and the anterior inferior cortex defined the ventral anterior insula (vAI). Division of the insular cortex into sub-regions based on anatomical features rather than clustering based on functional connectivity profiles was chosen to allow discussion of results based on known anatomical/histological features (Fig. 1).

### 2.6 Image processing and analysis

FSL5 software was used for processing of MRI data (FMRIB Software Library, Oxford, England; *http://www.fmrib.ox.ac.uk.fsl*). Pre-processing was performed, based on our previous pipeline (Hepp et al., 2017), using SIENAX on structural images and included brain extraction and brain volume calculation (normalized for head size) as well as grey- and white matter segmentation. Non-linear registration (FNIRT) was used to calculate registration parameters to standard space, which were inverted to bring the Automated Anatomic Labeling atlas (AAL) into subject anatomical space, using nearest neighbor interpolation. Insular nodes from the AAL were removed and replaced by Hammers_mith insular atlas. In order to avoid any overlap between the Hammers-mith insular atlas and the AAL atlas, we first merged and binarized the Hammers-mith atlas and used this mask to remove any overlapping voxels from the AAL atlas on a subject by subject basis. Afterwards, we added the insular mask including all sub-regions (non-binarized) to the final AAL atlas. The final AAL atlas including insular mask from the Hammers-mith atlas was then combined with FIRST-derived deep grey matter regions to form a whole-brain atlas. The full atlas was then multiplied with the SIENAX grey matter mask, to remove CSF and eliminate displacement due to grey matter atrophy, and registered to functional subject space using inverted boundary based registration (BBR) parameters and nearest neighbor interpolation.

Functional images were motion corrected, smoothed, high-pass filtered (100-second cutoff) and registered to standard space with the MELODIC pipeline using BBR as well as non-linear registration. Motion was measured as relative displacement which did not exceed 0.3 mm and was not significantly different between PD patients and controls (mean: 0.09 mm ± 0.05 and 0.10 mm ± 0.07 respectively; U = 419; *p* = 0.93) as previously described (Hepp et al., 2017). After registering the full atlas to fMRI space, mean functional time series were extracted for each individual grey matter region. Insular sub-regional connectivity patterns with the resulting brain regions were determined using a Pearson’s correlation matrix in Matlab 2012a (MathWorks, Inc., Natick, Massachusetts, United States). Five regions were excluded from final analysis due to geometric distortions, namely: the bilateral nucleus accumbens, rectal gyrus, olfactory gyrus, superior orbital frontal gyrus, and middle orbital frontal gyrus. Subsequently, connectivity matrices were reduced by averaging the two hemispheres and only including connections between the three insular sub-regions and the rest of the brain. These connections were then clustered (averaged) into connections with resting-state networks based on previous literature. Specifically, the AAL atlas and resting-state network parcellation maps provided by Yeo and colleagues were overlaid and each non-insular region in the AAL atlas was classified as being part of only one resting-state network based on highest overlap with each resting state network map (Yeo et al., 2011). This resulted in seven networks: visual, sensorimotor, ventral attention, limbic, fronto-parietal (FPN), deep grey matter, and default mode (DMN) networks; while the dorsal attention network was omitted due to the lack of sufficient number of regions forming the network (Supplementary-Table 1). The resulting correlation matrices represented connections between the three insular sub-regions and each of the seven resting-state networks, resulting in a 3 × 7 matrix for each subject. After creating this connectivity matrix, insular connectivity scores were averaged across hemispheres, due to the lack of a priori hypothesis on laterality in the insula and in order to reduce the number of tests for multiple comparisons.

### 2.7 Network importance: betweenness centrality

The Brain Connectivity Toolbox (BCT, https://sites.google.com/site/bctnet/) was used to calculate betweenness centrality (BC), which provides an estimate of the fraction of shortest paths travelling through a node, a metric measuring the relative importance of a node within the network (Rubinov and Sporns, 2010). The aforementioned whole-network weighted connectivity matrix was used, changing negative connections and the network diagonal to 0. The matrix was then converted to length measurements by inversion (1./X); the final BC metric was normalized through division with the maximum number of nodes to provide values ranging from 0 to 1. Normalized BC values were averaged to form mean BC values of insular sub-regions and networks. BC was only explored in regions showing significant changes in connectivity.

### 2.8 Statistical analysis

Normally distributed continuous demographic and clinical data were assessed using an independent two-sample *t*-test. Categorical variables were analyzed using Chi-square test. Our study design entailed correlation of network variables with CAMCOG scores followed by a linear regression model assessing predictors of cognitive performance, GLM for group comparisons of insular connectivity-regional differences, and finally BC of relevant network variables and relationship with CAMCOG. Correlations between network variables and other cognitive tests were also performed. Connectivity values between insular sub-regions and resting-state networks were correlated to CAMCOG scores, stratified per group, using Pearson’s partial correlation coefficients controlling for age, gender, and education (p < 0.05). This approach was also used for correlations with other cognitive tests with Bonferroni corrections for multiple comparisons. Network connections showing significant CAMCOG correlations were entered into a hierarchical (backward) linear regression model including age, gender, education, and BDI, to control for the effect of depression, to identify the most important cognitive correlates in each group. LEDD was also added as a covariate for PD group analyses. To limit the amount of statistical tests, a GLM was performed for all network regions to assess connectivity changes between groups. GLM analysis of between group differences for dAI and ACC was performed using bootstrapping. Normalized BC values showed a non-Gaussian distribution and were analyzed using non-parametric Mann-Whitney *U* test and Spearman’s Rho for correlations. A two-tailed *p* value threshold < 0.05 was used to indicate statistical significance. Correlations and post hoc comparisons between groups were performed with a Bonferroni correction for multiple group comparisons. All analyses were performed using SPSS 22.0 (*IBM, Chicago, Ill*). Mapping of network models of DMN-Insular and FPN-Insular FC were constructed using BrainNet viewer.

## 3 Results

### 3.1 Subject Demographics and clinical characteristics

There were no significant differences in sex distribution, age (Range: 52–81 years), or educational levels (Range: 2–6) between PD patients and controls (Table 1). Disease duration (Mean: 11.3 years (±3.6)) and disease severity (H&Y range: 2–3 and UPDRS III range: 14–56) were calculated for PD patients. CAMCOG scores were significantly lower in PD patients compared to controls (t(63) = 4.54, *p* < 0.001, d = 1.36) (Supplementary – Fig. 1). Exclusion of PD patients with CAMCOG total scores<80 (N = 6), indicative of dementia, also yielded significantly lower scores in PD (t(45) = 3.6, *p* = 0.001), while MMSE scores were not significantly different between groups (t(65) = 0.91, *p =* 0.336). For CAMCOG sub-scores, PD patients scored significantly lower than controls in the domains of language, memory, praxis, abstract thinking, and perception (*p* ≤ 0.01). BDI and BAI test scores, as well as scores on additional cognitive tests, i.e. semantic fluency, PRM, SSP, SWM, IED, and VPT were all significantly worse in PD compared to controls (*p* < 0.01).

### 3.2 Insular network connectivity and cognition

**Fig. 2:**
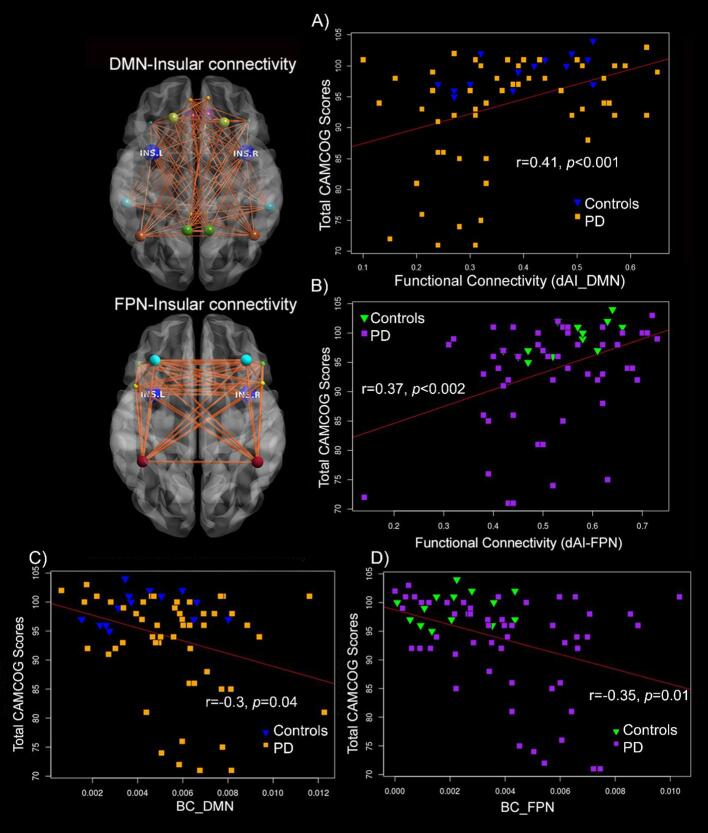
FC of dAI, DMN and FPN is important for cognitive performance. FC of dAI with DMN (r = 0.41, p < 0.001) and dAI with FPN (r = 0.37, p < 0.002) significantly correlated with Total CAMCOG scores across both groups (A-B). BC of both the DMN and FPN were higher in PD and negatively correlated with CAMCOG scores in PD only (controls are shown for reference) (C-D). BC: Betweenness Centrality; dAI: dorsal anterior insula; DMN: Default mode network; FC: functional connectivity; FPN: fronto-parietal network; INS.L: left insula; INS.R: right insula; PD: Parkinson’s disease.

Partial correlations between insula-network FC and CAMCOG across both groups combined showed significant results for several networks, after Bonferroni correction, including: dAI with sensorimotor network (r = 0.35, 95% BCa CI [0.1,0.55], *p* = 0.004), DMN (r = 0.41, 95% BCa CI [0.21,0.6], *p* = 0.001), FPN (r = 0.37, 95% BCa CI [0.08,0.58], *p* = 0.002), and ventral attention network (r = 0.33, 95% BCa CI [0.04,0.55], *p* < 0.007) (Fig. 2). These significant network variables were chosen and then placed in a backward regression model along with age, gender, education, LEDD, and BDI, controlling for the effects of depression, to identify which parameters most strongly related to CAMCOG performance in each of the groups. Since BAI scores were only available in a subsample of patients and showed strong correlations with BDI, we only included BDI in the analysis. In the control group, FC between the dAI and FPN was the significant predictor (β = 0.7, *p* = 0.003) and the model explained 50% of variance in CAMCOG scores (adjusted R^2^ = 0.5, F(1,13) = 13.50, *p* = 0.003). In the PD group, FC of dAI with DMN (β = 0.36, *p* = 0.002), age (β = −0.4, *p* = 0.002), BDI (β = −0.34, *p* = 0.004) and gender (β = −0.28, *p* = 0.02) were significant predictors of CAMCOG scores. The overall model (adjusted R^2^ = 0.37, F(4,51) = 8.5, *p* < 0.001) explained 37% of variance in CAMCOG.”

To verify whether the finding of dAI-DMN connectivity was driven by demented cases, this final model was repeated after excluding PD patients with CAMCOG scores < 80 (N = 6), i.e. using the significant predictors from the previous analysis in an “enter” model. This resulted in age (β = −0.5, *p* < 0.001) and dAI-DMN (β = 0.3, *p* = 0.016) as significant predictors in PD. The model (adjusted R^2^ = 0.35, F(4,41) = 7, *p* < 0.001) still explained 35% of variance.

### 3.3 Insula-DMN connectivity in PD

**Fig. 3:**
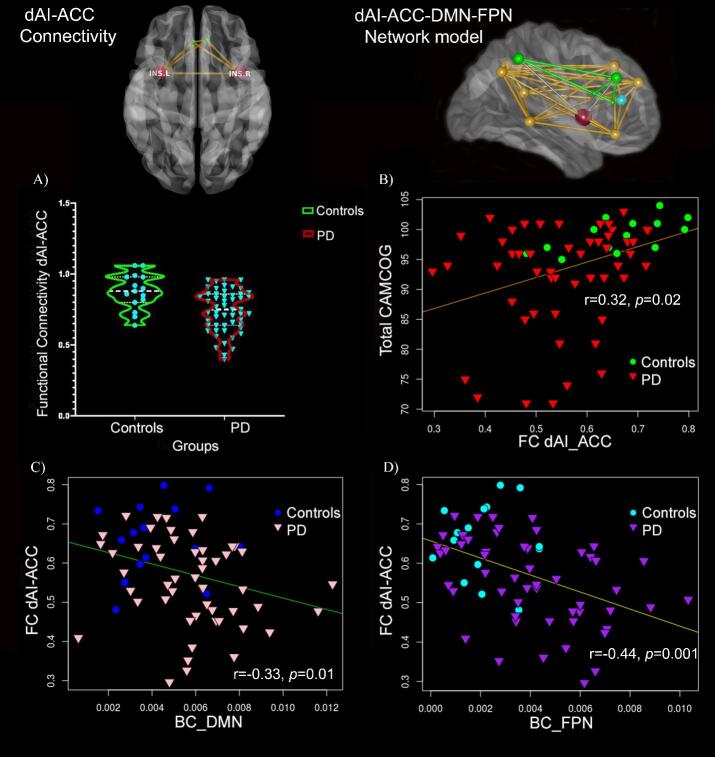
Reduced FC between the dAI and ACC in PD. FC between dAI and ACC was significantly reduced only in PD and significantly correlated with CAMCOG (A-B). BC of DMN and FPN negatively correlated with dAI-ACC FC in PD only (C-D). Controls are shown in the figure only for reference. Network models of connectivity between dAI and ACC (left) and between dAI, ACC, FPN, and DMN (right) are shown. ACC: anterior cingulate cortex; dAI: dorsal anterior insula; DMN: Default mode network; FC: functional connectivity; FPN: Frontoparietal network; INS.L: left insula, INS.R: right insula; PD: Parkinson’s disease.

Connectivity between the dAI and the DMN was subsequently explored further in PD by assessing FC changes between the dAI and regions composing the DMN (i.e. anterior cingulate, posterior cingulate, frontal superior, precuneus, middle temporal gyrus, angular gyrus, frontal inferior orbital gyrus, or frontal superior medial cortices). The GLM model showed a significant decline in FC between the dAI and the anterior cingulate cortex (ACC) in PD patients compared to controls (F(1,65) = 11, *p* = 0.002). FC between the dAI and the ACC showed a significant positive correlation with CAMCOG scores for both groups combined (r = 0.4, *p* = 0.001) and only a significant positive correlation in PD when comparing groups (r = 0.3, *p* = 0.027) (Fig. 3).

### 3.4 Insular-network connectivity and disease-specific measures in PD

Partial correlations between LEDD, UPDRS-III, H&Y scores and dAI-DMN as well as dAI-FPN FC did not reveal any significant results (p > 0.05). A positive correlation was found between disease duration and dAI-DMN FC (r = 0.34, *p* = 0.015). Other correlation analyses showed insignificant results after Bonferroni correction.

### 3.5 Betweenness centrality (BC)

BC was calculated for all 97 nodes, but only insular sub-regions as well as the DMN and FPN were compared between groups. Mann-Whitney *U* test showed significantly increased BC in PD versus controls for bilateral dAI (U = 488.5, *p* = 0.014), DMN (U = 5486, *p* = 0.015), and FPN (U = 492, *p* = 0.01). Post-hoc comparison of right and left dAI nodes showed significant group differences only for the right dAI (U = 509, *p* = 0.005). Only in PD, BC of the DMN (r_s_ = −0.3, 95% BCa CI [−0.58,−0.04], *p = 0.025*), and FPN (r_s_ = −0.35, 95% BCa CI [−0.62,−0.08], *p = 0.01*) correlated with CAMCOG total scores (Fig. 2). DMN and FPN BC were significantly negatively correlated with dAI-ACC FC in PD (r_s_ = −0.33, 95% BCa CI [−0.54,−0.92], *p* = 0.014; r_s_ = −0.44, 95% BCa CI [−0.65,−0.18], *p* = 0.001 respectively) (Fig. 3). In addition, assessment of correlation between BC and other clinical measures showed a significant positive correlation for DMN BC with BDI-based depression scores (r_s_ = 0.44, 95% BCa CI [0.17,0.65], *p* = 0.005).

## 4 Discussion

In this study, FC between the dAI and the DMN as well as FPN highly correlated with CAMCOG cognitive test scores. A shift in networks contributing to cognitive control was observed: whereas FC of the dAI with the FPN was associated with cognitive performance in controls, FC between the dAI and the DMN was associated with cognitive impairment in PD. Post-hoc analyses showed reduced FC between the anterior cingulate cortex (ACC) and dAI in PD. BC was significantly increased in PD for the right dAI, DMN, and FPN and negatively correlated with total CAMCOG scores for the latter two networks. In PD only, BC of DMN and FPN negatively correlated with FC of dAI with ACC. BC change in DMN was also related to depression in PD.

### 4.1 The dorsal anterior insula (dAI) and integration

First, our study shows that the dAI is particularly related to cognitive performance in PD. This is in line with other studies indicating a particular role of the dAI in higher cognitive as well as executive control (Deen et al., 20112011, Chang et al., 2013). The dAI has a complex transitional architecture, from allocortical to isocortical, and is in turn connected to various limbic and neocortical regions such as the orbitofrontal gyrus, olfactory cortex, cingulate gyrus, and parietal cortex (Mesulam and Mufson, 1982). Besides cognitive functions, the dAI appears to sub-serve socio-emotional, olfacto-gustatory, and sensorimotor functions, suggestive of its particular integrative functions (Kurth et al., 2010). In a recent functional meta-analytic study of the insular sub-divisions, it was shown that the dAI is a central co-activation region active across several functions and was proposed to play a role as an important functional brain hub (Uddin et al., 2014). Moreover, our centrality results show a stronger effect for the right dAI. This is in line with other studies showing that the right anterior insula is particularly involved in cognitive deficits in neurodegenerative diseases (Fathy et al., 2019).

### 4.2 The network triad and cognitive control

Second, our results indicate that of all the dAI connections, FC between the dAI and the DMN is strongly related to cognitive impairment in PD. The DMN has long been known for its role in spontaneous internal cognitive processes such as mind wandering and self-reflection. During externally induced goal-directed tasks, the DMN is deactivated (Raichle, 2015). In line with our results, reduced FC between regions composing the DMN was shown to correlate with lower cognitive performance, while reduced DMN FC with networks involved in attention underlie changes in executive processes in PD patients (Lucas-Jimenez et al., 2016). The FPN, also called the central executive network (CEN), is hypothesized to function as a control system governing several other distributed systems such as the DMN to control internal and external goal-directed cognitive processes (Dixon et al., 2018). In our study, connectivity between the dAI and FPN was the most important correlate of cognition in controls only, indicating a shift in networks contributing to cognitive control in disease. Notably, the dAI FC with FPN and DMN and their relationship with CAMCOG was not influenced by dopaminergic drug dosages (i.e, LEDD scores).

### 4.3 Anterior insula and cingulate Cortices: Histology, Pathology, and function

**Fig. 4:**
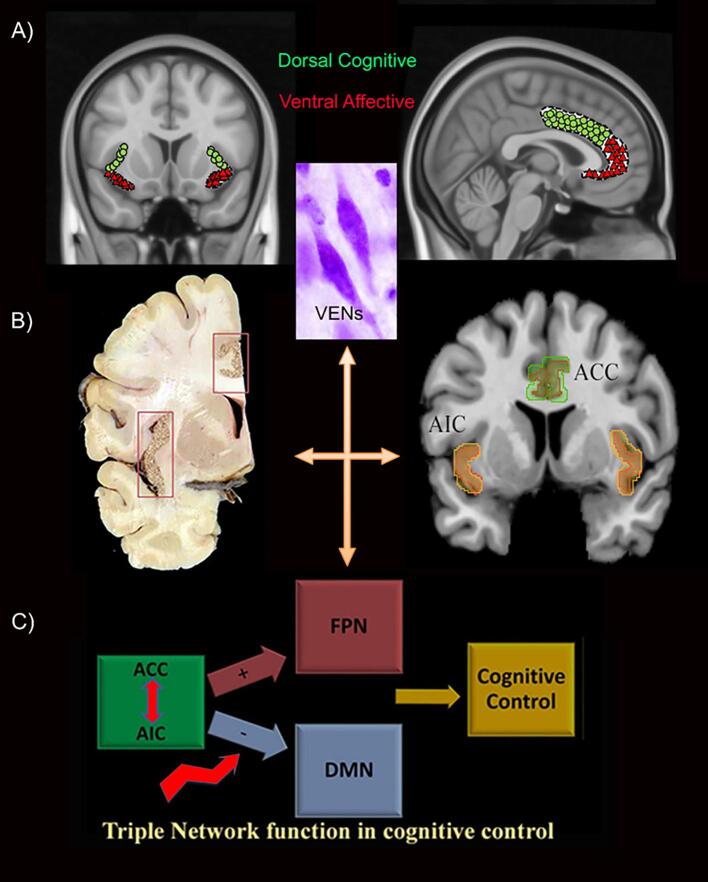
Structural and functional similarities between the ACC and AIC and role in cognition. (A) Both regions are sub-divided into dorsal cognitive and ventral affective sub-regions and contain unique von Economo neurons playing a role in awareness. (B) The insula and cingulate both show Lewy pathology during the same pathological stage (Braak stage 5) and control cognition through co-activation as a salience network whereby they can switch between other networks depending on task. (C) Disturbance of the network triad due to disconnection between AIC and ACC and altered centrality in DMN and FPN may lead to cognitive impairment in PD. ACC: Anterior cingulate cortex, AIC: Anterior insular cortex, DMN: Default mode network, FC: functional connectivity, FPN: Frontoparietal network.

The ACC was the primary driver for the reduced connectivity between the dAI and DMN and was also related to altered BC. The anterior insula and ACC share common histological features among which the presence of von Economo neurons (VENs). The VENs are sparsely branched neurons thought to play a role in social awareness and allow fast communication between both regions, which are similarly sub-divided into ventral affective and dorsal cognitive sub-regions (Menon and Uddin, 2010, Craig, 2009). In PD, both the insular and cingulate cortices show Lewy body (LB) pathology in advanced Braak LB stages V-VI, in which patients are thought to suffer from cognitive impairment (Del Tredici and Braak, 2016). Further assessment of insular pathological features showed more severe anterior insular LB pathology, including VENs, in PD (Fathy et al., 2019). The anterior insular cortex (AIC), believed to function as a core region in the salience network, along with the ACC, is proposed to control cognition by mediating a shift between DMN and FPN depending on the task (Fig. 4) (Sridharan et al., 2008, Greicius et al., 2003, Seeley et al., 2007). This process may be disrupted in PD, as evidenced by our results where dAI-ACC FC significantly correlated with increased centrality of DMN and FPN in PD, and poorer cognition. The dAI-ACC disconnection may disrupt the triple network function and result in a loss of normal mediation of cognitive control (Menon, 2011).

### 4.4 Limitations

Although this is the first study that assesses the functional connectivity of the insular sub-regions in PD, there are several limitations to consider. First, the study has a limited sample size, particularly for controls, which could limit the generalization of the results, especially with regards to effects of normal aging. Larger cohorts should further explore these effects as well as a more in-depth exploration of the effects of dementia. Second, we used a distinct network classification method that classifies each brain region as being part of only one network based on the highest overlap with the parcellation map by Yeo and colleagues (Yeo et al., 2011). This method, although well characterized and embedded in current literature, may be less accurate compared to the data-driven independent component analysis approach to define networks. However, this was specifically chosen because it was determined in a very large sample of controls, while ours was limited. Moreover, we used an anatomical rather than functional insular atlas, although both classifications are highly similar, to allow optimal comparison with histopathological studies. In this study, our analyses were limited to the aforementioned atlases, given the limited power, yet future studies could explore voxel-wise changes in more detail. In addition, distortion effects could have impacted our results, although the exclusion of regions was minor. Regarding global network changes, our a-priori hypothesis was that the flow of information between the DMN, FPN, and dorsal insula has changed in PD, thus we chose BC as a sensitive centrality measure, assessing changes in the shortest paths between regions. Other network measures, however, could also be explored in future work. Moreover, for this study, our primary aim was to assess insula-network FC in relation to cognitive performance and to further dissect the circuitry responsible for impairment in PD, therefore, we did not assess group differences in network connectivity. Last, we have only included patients in the “ON” medication state. This was done to avoid uncontrolled head motion that may have effects on the BOLD signal particularly for our cohort with moderate disease severity and disease duration. In addition, evidence shows that patients scanned in the “OFF” medication state may still have residual effects from chronic treatment compared to de Novo patients who have not received any treatment (Tahmasian et al., 2015). Although we used LEDD as a covariate, careful interpretation of the results remains important as medication effects on functional connectivity cannot be ruled out. We believe that future studies should include diffusion tensor imaging data to dissect structural connectivity changes in PD, particularly those related to the dAI.

## 5 Conclusions

In this study, FC of the dAI sub-region of the insula, particularly connectivity of the dAI with the DMN, was most important in explaining cognitive performance in PD. Notably, FC between dAI and ACC was reduced in PD and correlated with cognitive performance. While, DMN and FPN showed increased centrality which negatively correlated with cognition as well as reduced connectivity between the dAI and ACC in the PD group. Our study therefore indicates that the anterior insular cortex, a known network connector hub, is involved in PD-related cognitive impairment, possibly through destabilization of the DMN and FPN induced by loss of connectivity with the ACC. Future studies ought to pinpoint the histopathological underpinnings of these functional changes.

## CRediT authorship contribution statement

**Yasmine Y. Fathy:** Conceptualization, Methodology, Formal analysis, Writing - original draft, Writing - review & editing, Visualization. **Dagmar H. Hepp:** Methodology, Writing - original draft, Writing - review & editing. **Frank J. de Jong:** Writing - original draft, Writing - review & editing, Funding acquisition. **Jeroen J.G. Geurts:** Writing - original draft, Writing - review & editing. **Elisabeth M.J. Foncke:** Writing - original draft, Writing - review & editing, Resources. **Henk W. Berendse:** Conceptualization, Resources, Writing - original draft, Writing - review & editing, Supervision. **Wilma D.J. van de Berg:** Conceptualization, Writing - original draft, Writing - review & editing, Funding acquisition. **Menno M. Schoonheim:** Conceptualization, Methodology, Writing - original draft, Writing - review & editing, Resources, Supervision.

## Declaration of Competing Interest

The authors declare that they have no known competing financial interests or personal relationships that could have appeared to influence the work reported in this paper.
