# Supplementary material for: Anterior insular network disconnection and cognitive impairment in Parkinson’s disease
Source: Neuroimage Clin. 2020 Jul 25;28:102364. doi: 10.1016/j.nicl.2020.102364 (PMC7417948; doi:10.1016/j.nicl.2020.102364)
Supplement: Supplementary data 1 [file mmc1.docx]

**Supplementary**

**Anterior insular network disconnection and cognitive impairment in Parkinson’s disease**

Yasmine Y. Fathy MD^1,3*^, Dagmar H. Hepp MD,PhD^2^, Frank J. de Jong MD,PhD^3^, Jeroen J.G. Geurts PhD^1^, Elisabeth M.J. Foncke MD,PhD^2^, Henk W. Berendse MD,PhD^2^, Wilma DJ. van de Berg PhD^1^, Menno M. Schoonheim PhD^1^

^1^ Department of Anatomy and Neurosciences, Amsterdam UMC, Vrije Universiteit Amsterdam, Amsterdam Neuroscience, De Boelelaan 1108, 1081 HZ Amsterdam, Netherlands

^2^ Department of Neurology, Amsterdam UMC, Vrije Universiteit Amsterdam, Amsterdam Neuroscience, De Boelelaan 1117, 1081 HZ Amsterdam, Netherlands

^3^Department of Neurology, Erasmus Medical Center, Postbus 2040 3000 CA, Rotterdam, Netherlands

y.fathy@amsterdamumc.nl

[d.hepp@amsterdamumc.nl](mailto:d.hepp@amsterdamumc.nl)

f.j.dejong@erasmusmc.nl

[J.Geurts@amsterdamumc.nl](mailto:J.Geurts@amsterdamumc.nl)

[e.foncke@amsterdamumc.nl](mailto:e.foncke@amsterdamumc.nl)

H.Berendse@amsterdamumc.nl

[WDJ.vandeBerg@amsterdamumc.nl](mailto:WDJ.vandeBerg@amsterdamumc.nl)

m.schoonheim@amsterdamumc.nl

**Corresponding author:**Yasmine Fathy, MD
Dept. of Anatomy & Neurosciences
VU University Medical Center
O2 building, room 13 W01
De Boelelaan 1108
1081 HZ Amsterdam, Netherlands

e-mail: y.fathy@amsterdamumc.nl;
ORCID: 0000-0001-9236-2479

Keywords: Insular cortex, resting state fMRI, cognitive impairment, Parkinson’s disease, network triad

**Table 1. Network Composition**

| Networks | Regions |
| --- | --- |
| Visual | **Calcarine, Cuneus,_Fusiform gyrus,_Lingual gyrus, Superior Occipital gyrus, Middle Occipital gyrus, Inferior Occipital gyrus** |
| Sensorimotor | **Heschl gyrus, Paracentral Lobule, Precentral gyrus, Postcentral gyrus, Rolandic Operculum, Supplementary Motor Area, Superior Temporal gyrus** |
| Ventral attention | **Inferior Frontal Operculum, Middle Cingulate gyrus, Supramarginal gyrus** |
| Limbic | **Para-Hippocampal gyrus, Superior Temporal Pole, Middle Temporal Pole, Inferior Temporal gyrus** |
| Fronto-parietal | **Inferior Parietal gyrus, Middle Frontal gyrus, Inferior frontal triangular gyrus, Inferior frontal operculum** |
| Default mode | **Precuneus, Superior Frontal gyrus, Middle Temporal gyrus, Angular gyrus, Inferior orbito-frontal gyrus, Anterior cingulate gyrus, Superior Medial frontal gyrus, Posterior cingulate gyrus** |
| Deep grey matter | **Caudate nucleus, Putamen, Thalamus Proper** |

**Table 1. Network Composition**. Networks were classified based on highest overlap between the AAL atlas and parcellation maps provided by Yeo and colleagues. Bilateral brain regions are included in each network.

**
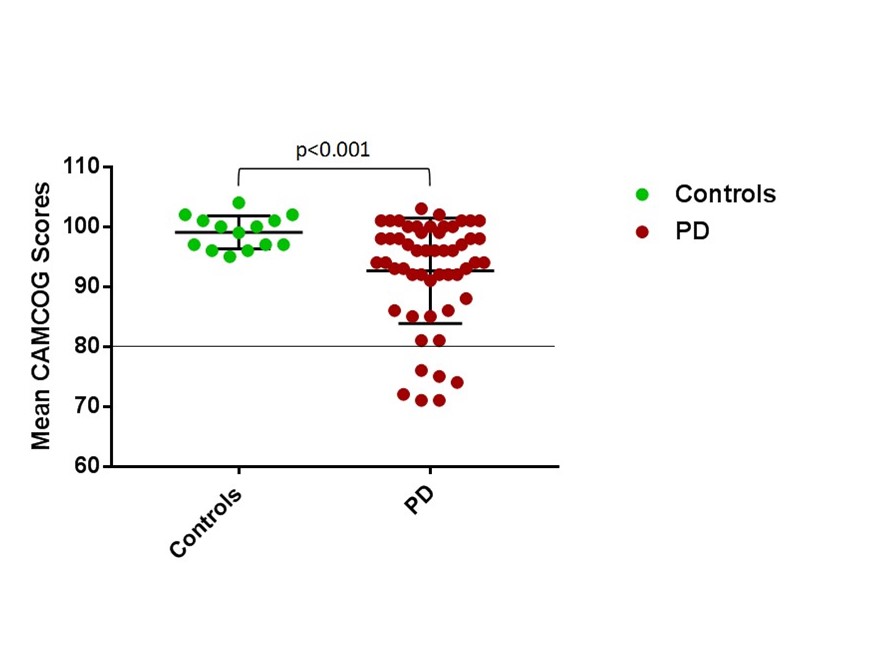
**

**Figure 1. Significantly reduced CAMCOG scores in PD compared to controls.** Mean total CAMCOG scores and standard deviation (bars) show significant differences between PD and controls (p<0.001). A score below 80 indicates dementia.
